# Comparison of The Results of Sponsored Genetic Testing Panels for Inherited Retinal Diseases

**DOI:** 10.3390/jcm13113118

**Published:** 2024-05-26

**Authors:** Yicheng K. Bao, Betty A. Situ, Margaret Runner, Andrew Moshfeghi, Hossein Ameri

**Affiliations:** 1 Department of Ophthalmology, USC Roski Eye Institute, Keck School of Medicine, University of Southern California, 1450 San Pablo Street, Los Angeles, CA 90033, USA; yicheng.bao@med.usc.edu (Y.K.B.); betty.situ@med.usc.edu (B.A.S.); andrew.moshfeghi@med.usc.edu (A.M.); 2 Retina Center of Texas, Dallas, TX 75243, USA; margaret.runner@retinacentertx.com

**Keywords:** inherited retinal disease, genetic testing, retinitis pigmentosa, Invitae, Blueprint Genetics

## Abstract

**Background/Objectives**: Gene therapy’s emergence has made molecular diagnosis for inherited retinal diseases clinically significant. Free genetic testing panels have improved testing access in clinical practice, yet the interpretation of results, especially variants of unknown significance (VUS), remains challenging and requires expertise. This study shares our experience in utilizing sponsored IRD panel tests by Invitae and Blueprint Genetics (BG), reporting their positivity rates, and comparing their reclassification of variants through amendments. **Methods**: This retrospective study analyzed genetic test reports from patients who underwent testing via Invitae or BG panels. A positive test was determined if there was a pathogenic mutation in an autosomal dominant gene, two pathogenic mutations in an autosomal recessive gene, or a pathogenic mutation in an X-linked gene in a male patient. **Results**: The testing positivity rates were 34.9% for Invitae (n = 109) and 42.1% for BG (n = 107). Invitae had more pathogenic variants per report (0.87 vs. 0.58 variants, *p* = 0.0038) and issued more amendments than BG (0.54 vs. 0.03 amendments; *p* < 0.01). Of the Invitae variant classification changes, 66.2% switched a VUS to benign. In the BG group, 75% of variant reclassifications changed a VUS to pathogenic. As a result of the Invitae amendments, 88% did not change the overall report result. **Conclusions**: While free-of-charge genetic testing panels offer valuable insights for diagnosing IRD, limitations such as low diagnostic yield and variant classification discrepancies persist between Invitae and BG. VUS should not be considered pathogenic in the clinical decision-making process. Careful interpretation of genetic testing is required.

## 1. Introduction

Inherited retinal diseases (IRDs) are a significant cause of blindness in the world. IRDs are characterized by monogenic defects in the molecular machinery of the neurosensory retina, retinal pigmented epithelium (RPE), or choroid that cause vision loss. Historically, there has been no cure for IRDs. The American Academy of Ophthalmology Guidelines previously recommended, in 2012, that ophthalmologists avoid routine genetic testing for IRD because a molecular diagnosis would not significantly change disease management [1]. However, the curative power of gene therapy has the potential to revolutionize the care of patients with inherited retinal diseases. The first gene therapy to gain regulatory approval was voretigene neparvovec-rzyl (Luxturna; Spark Therapeutics), used to treat RPE65-mediated retinal dystrophy [2]. There are numerous other gene therapies under investigation in current and planned clinical trials [3]. In the modern era of gene therapy, a molecular diagnosis has the potential to change management and provide a cure. Thus, since the approval of voretigene neparvovec-rzyl, two programs began offering free-of-charge genetic testing panels for IRDs: ID your IRD through Invitae (San Francisco, CA, USA), sponsored by Spark Therapeutics (Philadelphia, PA, USA); and My Retina Tracker through Blueprint Genetics (BG), sponsored by the Foundation Fighting Blindness (Columbia, MD, USA). Both programs provide panel genetic testing for a pre-specified list of genes known to cause IRDs. Of note, these do not provide whole genome sequencing or whole exome sequencing. Previous studies have found that a genetic diagnosis has been possible in 25–78.8% of cases using a panel approach [4,5,6].

These testing programs offered by Invitae and BG are now widely used in clinical practice due to their ease of access and lack of direct cost to the patient, physician, or payor. Invitae and BG provide a report for each patient containing a list of mutations in genes known to cause IRDs and a determination of whether the patient’s phenotype can be explained by one of these variants. These reports are also amended by Invitae and BG, where previously identified variants of unknown significance (VUS) may be reclassified as pathogenic or benign based on further analysis. Unlike many medical tests in which the results are self-explanatory, interpretation of genetic testing results requires some expertise. Since the sponsored panels can be utilized by any ophthalmologist or optometrist, many providers who are not familiar with genetic testing rely entirely on the interpretation provided in the genetic testing report. It is not uncommon to see that some providers and patients assume VUS is a disease-causing mutation. The purpose of this study was to share our experience in utilizing sponsored IRD panel tests by Invitae and BG, compare their reporting patterns, and determine how later reclassification of variants, particularly VUS, may change the interpretation of the tests. 

## 2. Materials and Methods

This retrospective study was approved by the University of Southern California (USC) Institutional Review Board (IRB) and adhered to the Declaration of Helsinki and the United States Health Insurance Portability and Accountability Act (HIPAA) of 1996. The study involved the review and analysis of genetic test reports of patients with a definitive, clinically diagnosed IRD or suspicion of an IRD from the USC Roski Eye Institute and Los Angeles General Medical Center (Formerly Los Angeles County + USC Medical Center) who underwent genetic testing from August 2019 to June 2023 utilizing sponsored IRD panels by Invitae or BG. 

Consecutive genetic testing reports were reviewed for variants and report results. Demographic factors such as age and gender were also recorded. When reviewing the reports, we determined the positivity of genetic testing based on variant classification only, not the report outcome. One pathogenic or likely pathogenic variant in an autosomal dominant gene was considered positive, and one pathogenic or likely pathogenic variant in an X-linked gene in a male was considered positive. For autosomal recessive genes, two pathogenic variants were required to be considered positive. One pathogenic or likely pathogenic variant in an autosomal recessive gene was considered a carrier. Similarly, one pathogenic or likely pathogenic variant in an X-linked gene in a female was considered a carrier. In cases where no pathogenic or likely pathogenic variants were found, this was considered a negative test. 

Categorical variables were compared using the Chi-squared test. Continuous variables were compared using the *t*-test. Data were analyzed using SAS Analytics, version 9.4 (SAS Institute, Cary, NC, USA). 

## 3. Results

The cohort included 216 total patients who underwent genetic testing; 109 patients underwent testing with the Invitae panel, and 107 patients underwent testing with the BG panel. A total of seven patients were tested by both laboratories. The mean age for the total cohort was 48.5 ± 17.1 years, 47.2 ± 17.4 years in the Invitae cohort, and 50.0 ± 16.7 years in the BG cohort (Table 1; *p* = 0.2207). There were 101 males in the total cohort (47.2%), 42 (38.5%) males in the Invitae group, and 59 (55.1%) males in the BG group (Table 1; *p* = 0.0165).

### 3.1. Pattern of Reporting

The Invitae and BG IRD panels include 330 and 351 genes, respectively. They have 280 genes in common, with 50 genes tested in the Invitae panel only and another 72 genes in the BG panel only. A list of all genes tested using both Invitae and BG are listed in Supplemental Table S1; a list of all the genes only tested using Invitae and not tested using BG, as well as a list of all the genes tested using BG and not using Invitae, are listed in Supplemental Table S2. The Invitae panel initially included 248 genes, which increased to 293 in late 2020 and, subsequently, to 330 in late 2021, at which time RPGR was added to the panel. The BG panel contains 314 nuclear genes and 37 mitochondrial genes. The Invitae genetic testing reports include a “Results” heading, labeling the test as “Positive”, “Potentially Positive”, “Carrier”, or “Uncertain”. They do not mention whether the clinical information provided in the test requisition order form is used for such determinations. The BG genetic testing reports provide a “Summary of Results”, which includes a “Primary Findings” subheading. They either state “Negative for explaining the patient’s phenotype” or they present variants that may be relevant to the patient’s phenotype. Invitae and BG classified the pathogenicity of these variants based on their own, separate proprietary algorithms [7,8,9]. As BG states on their website, “Despite the international guidelines and principles, there is still significant inter-laboratory variation in classification of genetic variants” [8]. After the initial genetic testing reports were released, both Invitae and BG sent electronic correspondence with amendments to the original genetic reports. These amendments reclassified the pathogenicity of variants based on new data. As a result of these variants, there was also potential for the overall report result to change, in which a negative genetic test could turn to a positive genic test. Invitae reported several patients as “Potentially Positive” in which there was a pathogenic mutation in an autosomal recessive gene and there was another VUS. For the purposes of this analysis, these patients were counted as negative because there was uncertainty whether the VUS was pathogenic and no segregation analysis was conducted to determine whether the other alleles came from separate chromosomes.

### 3.2. Overall Test Results

The total number of positive tests was 82 (38.0%) for the entire cohort; 37 (33.9%) in the Invitae group; and 45 (42.1%) in the BG group. The Invitae cohort had ten (9.2%) patients with autosomal dominant disease, twenty-six (23.9%) patients with autosomal recessive disease, one patient (0.9%) with X-linked disease, thirty-four (31.2%) patients who were carriers of IRD, and thirty-eight (34.8%) patients who were negative for disease causing mutations in the panel (Table 2). The BG cohort had sixteen (15.0%) patients with autosomal dominant disease, twenty-three (21.5%) patients with autosomal recessive disease, four (3.7%) with X-linked disease, two (1.9%) with mitochondrial disease, eighteen (16.8%) patients who were carriers of IRD, and forty-four (41.0%) patients who were negative for disease-causing mutations in the IRD panel (Table 2).

The most common genes with pathogenic or likely pathogenic variants, in the order of frequency, were *ABCA4*, *USH2A*, and *EYS* in the Invitae reports (Supplemental Table S3) and *ABCA4*, *RHO*, *EYS*, and *USH2A* in the BG reports (Supplemental Table S4). The most common genes in the Invitae group listed as VUS were *MYO7A*, *VPS13B*, *USH2A*, *ADGRV1*, *VCAN*, *CACNA2D4*, and *EYS* (Supplemental Table S3). The most common genes in the BG group listed as VUS were *USH2A*, *ABCA4*, *KIAA1549*, *EYS*, and *AGBL5* (Supplemental Table S4).

The mean number of pathogenic variants per patient was significantly higher in the Invitae group (0.87 ± 0.79 variants, ranging from 0 to 3) than the BG group (0.58 ± 0.68, ranging from 0 to 3); *p* = 0.0038). The mean number of VUSs per patient was significantly higher in the Invitae group (4.1 ± 2.5 (range 0–11)) than the BG group (1.5 ± 1.3 (range 0–5); *p* < 0.0001. 

One patient in the Invitae group was a carrier for a gene (*CLN8*) that was not tested for in the BG panel. Two patients in the BG group had pathogenic mutations in *MT-ATP6* and *MT-ND1*, which were not tested for in the Invitae panel, and one patient was a carrier in *MMAHC*, which was not tested for in the Invitae panel.

### 3.3. Amendment and Variant Reclassification

The total number of amendments was fifty-nine in the Invitae group and three in the BG group. The mean number of amendments per patient was 0.54 ± 0.80 in the Invitae group and 0.03 ± 0.17 in the BG group. In the Invitae group, twenty-three patients had one amendment, fifteen patients had two amendments, and two patients had three amendments. In the BG group, three patients had one amendment, and there were no patients with more than one amendment. Some amendments changed the classification of multiple variants. The Invitae group had seventy-one variant classifications changed in fifty-nine amendments, while the BG group had four variant classifications changed in three amendments. 

The mean time to amendment was greater for the BG group (655.7 ± 224.1 days (range: 427–875 days)) than the Invitae group (600.3 ± 362.8 days (range: 48–1255 days); *p* < 0.0001). Of the Invitae variant reclassifications, 66.2% switched a VUS to benign, 21.1% changed a VUS to pathogenic or likely pathogenic, 11.3% switched likely pathogenic to pathogenic, and 1.4% switched benign to VUS (Table 3). In the BG group, 75% of the variant reclassifications changed a VUS to pathogenic, and 25% of amendments changed a likely pathogenic to pathogenic (Table 3). As a result of the Invitae amendments, fifty-two (88.1%) of the amendments did not change the overall report result, two (3.4%) of the amendments changed a negative result to a carrier, and five (8.5%) of the amendments changed a negative result to a positive one. As a result of the BG amendments, two out of the three amendments in the BG changed the overall report result from negative to positive. One out of three amendments in the BG group did not change the overall report result.

### 3.4. Test Results of Patients Tested with Both Invitae and BG

This cohort had seven patients tested with both kits from Invitae and BG. Patient one had a clinical diagnosis of cone dystrophy, and the Invitae kit reported VUS in *ARHGEF18*, *INPP5E*, *NPHP3*, and *SEMA4A*; BG reported VUS in *ARHGEF18* and *SEMA4A*. All four of these genes are included in both panels. Patient two had a clinical diagnosis of retinitis pigmentosa and the Invitae kit reported *BBS10*, *CA4*, and *NPHP1* as VUS; BG reported *RCBTB1* and *CA4* as VUS. All four of these genes are included in both panels. Patient three had a clinical diagnosis of macular dystrophy, however, Invitae and BG reported different pathogenic variants. Invitae reported that this had a pathogenic deletion in *CLN3*, which is associated with autosomal recessive retinitis pigmentosa; BG also reported this *CLN3* allele as pathogenic. However, BG reported this patient to have a pathogenic deletion in *CRX*, which has been associated with autosomal dominant retinitis pigmentosa. Interestingly, Invitae reported this *CRX* deletion as a VUS. The BG report stated this patient had positive genetic testing due to the *CRX* deletion. Instead, the Invitae report stated this patient was just a carrier for *CLN3* due to its autosomal recessive inheritance pattern and there was no causative genetic diagnosis for the patient’s phenotype. Both *CRX* and *CLN3* were included in both panels. Patient four had a clinical diagnosis of retinitis pigmentosa and Invitae reported this patient to have a pathogenic mutation in *CLN8*, which is associated with autosomal recessive retinitis pigmentosa; however, *CLN8* is not included in the BG panel. BG reported a pathogenic mutation in *VPS13B*, which is associated with Cohen syndrome, but stated this mutation did not explain the patient’s phenotype. Despite the fact that the Invitae panel included screening for variants in *VPS13B*, the report did not mention any pathogenic mutation or VUS for this gene. Patient five had a clinical diagnosis of posterior polar choroidal annular dystrophy and both Invitae and BG found a likely pathogenic mutations in *SAG*, which is associated with autosomal dominant retinitis pigmentosa and autosomal recessive Oguchi disease. However, BG detected an additional pathogenic variant in *TTPA*, but Invitae reported this as a VUS. Both *SAG* and *TTPA* are included in both panels. Patient six had a clinical diagnosis of retinitis pigmentosa with vasoproliferation. Invitae reported VUS in *BBS12*, *CEP164*, *CLN3*, *COL18A1*, *TUBGCP6*, *USH1G*, and *USH2A*. BG reported VUS in *BBS12*, *CLN3*, and *USH2A*. All seven of these genes are included in both panels; however, no mention of VUS in *CEP164*, *COL18A1*, *TUPGCP6*, and *USH1G* was made in the BG panel. Patient seven had a clinical diagnosis of cone–rod dystrophy. Invitae determined this patient to have a pathogenic mutation in *TMEM231*, which has been associated with autosomal recessive Joubert syndrome. However, BG made no mention of this allele, and only reported various VUSs in *ABCA4*, *ATF6*, and *KCNV2*. All four of these genes were included in both panels.

## 4. Discussion

Free-of-charge sponsored genetic testing panels can provide genetic diagnoses to patients with clinical IRDs. Our cohort was positive for IRD in 38.4% of patients, which is similar to prior reports [4,5,6]. However, prior reports have not described amendments issued by the laboratories nor the effects of these amendments on the overall report result. Our study has found that Invitae was significantly more likely to issue amendments than BG and that 88.1% of the amendments did not change the overall report result. Our study also found 76% of VUS that were later defined in a follow-up amendment to be benign. As such, physicians, patients, and genetic counselors should be cautious about assuming VUS are pathogenic in cases that fit the clinical picture, as they are more likely to be reported to be benign than pathogenic in follow-up amendments. Both Invitae [10,11] and BG [12] provide some documentation about their process to classify variants. Invitae uses a novel population allele frequency model to estimate the probability of a particular allele to be pathogenic or benign [13]. Interestingly, as BG states on their website, “Despite the international guidelines and principles, there is still significant inter-laboratory variation in classification of genetic variants” [8]. This reinforces the subjective nature of variant classification. Ultimately, it is at the discretion of each laboratory to issue amendments and classify VUS at their own pace. The American College of Medical Genetics and Genomics and the Association for Molecular Pathology guidelines state that VUS should not be used in clinical decision making [14]. Our study corroborates this guideline given that the majority of VUS are determined to become benign. Given that each laboratory has a separate process for classifying VUS, it is likely that some VUS classifications would differ between labs.

It is imperative that physicians and patients alike understand that these free-of-charge genetic testing panels are limited to a certain subset of genes. Approximately 71.8% of inherited retinal diseases are attributed to only 20 genes [15]. Thus, it can be expected that a reasonable number of patients with a genetic cause of IRD can be detected using a genetic testing panel that examines a limited number of genes. The current approach to free-of-charge genetic panel testing for IRD is likely the most economical, given the high percentage of IRDs caused by a limited number of genes. However, genes that are not included in the panel are missed; in some cases, the patients’ IRD clinical phenotype cannot be explained by presently described genes and alleles. For example, *PGK1* (phosphoglycerate kinase) can cause a phenotype of retinitis pigmentosa and myopathy [16]. However, this gene was not included in either panel. Furthermore, there are many variants in known, IRD-causing genes that have not been classified in terms of pathogenicity, which are VUSs [14]. In addition, non-coding elements, such as transcription factors and cis-regulatory elements, also exert regulatory effects on genes that could be causative for retinal disease phenotypes [17]. The BG panel captures one hundred twenty-nine non-coding variants across forty-nine IRD genes; the Invitae IRD panel captures three non-coding variants across two genes [18]. Furthermore, the Invitae panel does not test for any mitochondrial genes. Physicians, patients, and genetic counselors must be aware of the limitations of these targeted panels in detecting variants in non-coding sequences that could be causing clinical IRDs.

The differences in variant detection results for our cohort of patients tested by both companies are striking. There was discordance between Invitae and BG in their variant detection and classification in half of patients who were tested by both: patient three, patient four, and patient seven. A *CRX* deletion was not detected by Invitae in patient three; a pathogenic *CLN8* mutation was reported by Invitae, but this gene was not included in the BG panel for patient four; and a mutation in *TMEM231* was not detected by BG in patient seven. Both Invitae and BG reported using next-generation sequencing to detect variants. Invitae reports an average depth coverage of 350× (Ref. [19]), and BG reports an average sequencing depth of 143× [20]. Differences in sequency depth could be a contributor, but these are likely adequate. Another potential cause of discordance could be differences in the algorithms used for aligning sequences to the reference genome and variant calling. In addition, each company uses different proprietary variant databases, leading to differences in variant classification. Although both companies are using the American College of Medical Genetics and Genomics and the Association for Molecular Pathology criteria, interpretation of these criteria could have a subjective element leading to discordance in variant classification. Given that genetic knowledge is rapidly evolving, companies update their variant databases at different frequencies based on new information in the literature, which could then lead to different variant classifications. A small possibility is that differences in sample handling, quality, and processing may also contribute to variations in results due to high modern standards for sample handling and processing. However, this would more likely explain individual discrepancies rather than systemic differences between the laboratories. High-quality, well-preserved DNA samples are crucial for accurate genetic testing, and any degradation or contamination can affect the outcome. Thus, we recommend that physicians, patients, and genetic counselors interpret any single test with caution.

This phenomenon has been seen elsewhere when testing for ancestry among identical twin pairs, in which there was only 34.1% concordance for the deep sleep trait among twin pairs when tested by different companies [21]. These results demonstrate the limitations of the genetic testing panel approach, because each genetic testing lab screens a select subset of genes and uses different methods for variant classification. In addition, each lab uses expert opinions from geneticists and physicians to ultimately determine allele pathogenicity and there is a component of subjectivity, which is stated by the laboratories. This has large implications in clinical practice, in that one genetic testing panel may be insufficient to reach a molecular diagnosis.

Gender is not considered a significant confounder in IRD testing, except for in the context of X-linked diseases. There was a higher proportion of males tested using BG vs. Invitae (55.1% vs. 38.5%), which could have allowed BG to find a greater proportion of X-linked diseases than Invitae (3.7% vs. 0.9%). However, these findings do not bias the greater findings of this study.

Accurate and precise genetic testing for IRDs significantly impacts patient management and outcomes by providing precise molecular diagnoses that can guide clinical decisions. Most importantly, identifying genetic variants allows for corrective therapy with gene therapy, including CRISPR (Clustered Regularly Interspaced Short Palindromic Repeats) and AAV (Adeno Associated Virus)-mediated gene replacement. In addition, genetic testing informs surveillance strategies by identifying patients at risk for associated systemic conditions, enabling proactive monitoring. Prognostic information derived from genetic testing helps in counseling patients and their families about disease progression and the probability that their offspring will be affected by a particular disease, facilitating informed decision-making regarding family planning. Thus, accurate genetic testing results have the power to enhance personalized care, optimize therapeutic strategies, and improves overall patient outcomes in IRDs.

Given the low diagnostic yield and the complexity of genetic test interpretation, genetic testing kits should not be utilized in clinical practice as screening tools in cases with a low suspicion of an IRD. The ability of one of these genetic testing kits to rule in or rule out a diagnosis is poor given the diagnostic yield of 34.9% in the Invitae group and 42.1% in the BG group. The high rate of VUSs and uncertain results can also cause confusion to both the patient and physician. Notably, VUSs should not be considered pathogenic nor used in the clinical decision-making process.

In conclusion, sponsored genetic testing panels for IRDs have become a tremendous resource in clinical practice. However, significant limitations persist, such as low diagnostic yield and discordance in variant detection and classification between laboratories. Physicians and patients should be aware of these limitations and exercise caution when interpreting these tests.

## Figures and Tables

**Table 1:** Cohort demographics.

	Total Cohort	Invitae	BG	*p*-Value
n	216	109	107	n/a
Age (years)	48.5 ± 17.1	47.2 ± 17.4	50.0 ± 16.7	0.2207
Gender (% Male)	101 (47.2%)	42 (38.5%)	59 (55.1%)	0.0165

**Table 2:** Inheritance pattern of positive genetic tests by laboratory.

	Invitae n (%)	Blueprint Genetics n (%)	Total Cohort n (%)
Autosomal Dominant	10 (9.2%)	16 (15.0%)	26 (12.0%)
Autosomal Recessive	26 (23.9%)	23 (21.5%)	49 (22.7%)
X-Linked	1 (0.9%)	4 (3.7%)	5 (2.3%)
Mitochondrial	Not Tested	2 (1.9%)	2 (0.9%)
Carrier	34 (31.2%)	18 (16.8%)	52 (24.1%)
Negative	38 (34.8%)	44 (41.0%)	82 (38.0%)

**Table 3:** Results of variant classification changes by laboratory.

	Invitae	Blueprint Genetics
Variant Classification Change	Frequency (%)	Frequency (%)
VUS to Benign	47 (66.2%)	0 (0%)
VUS to Pathogenic	14 (19.7%)	3 (75%)
VUS to Likely Pathogenic	1 (1.4%)	0 (0%)
Likely Pathogenic to Pathogenic	8 (11.3%)	1 (25%)
Benign to VUS	1 (1.4%)	0 (0%)

## Data Availability

The data presented in this study are contained within the article, except the actual genetic testing reports, which, due to patient privacy concerns, cannot be provided.

## Supplementary Materials

The following supporting information can be downloaded at: https://www.mdpi.com/article/10.3390/jcm13113118/s1, Table S1. List of Genes Tested by Both Invitae and Blueprint Genetics; Table S2. List of Genes Tested by Invitae Only and BG Only; Table S3. Frequency of Genes found to be pathogenic, likely pathogenic and VUS in Invitae Gene Panel; Table S4. Frequency of Genes found to be pathogenic, likely pathogenic and VUS in Blueprint Genetics Panel.

## References

[1] Stone E.M., Aldave A.J., Drack A.V., Maccumber M.W., Sheffield V.C., Traboulsi E., Weleber R.G. (2012). Recommendations for Genetic Testing of Inherited Eye Diseases: Report of the American Academy of Ophthalmology Task Force on Genetic Testing. Ophthalmology.

[2] Commissioner O. of the FDA Approves Novel Gene Therapy to Treat Patients with a Rare Form of Inherited Vision Loss. https://www.fda.gov/news-events/press-announcements/fda-approves-novel-gene-therapy-treat-patients-rare-form-inherited-vision-loss.

[3] Ameri H., Kesavamoorthy N., Bruce D.N. (2023). Frequency and Pattern of Worldwide Ocular Gene Therapy Clinical Trials up to 2022. Biomedicines.

[4] McClard C.K., Pollalis D., Jamshidi F., Kingsley R., Lee S.Y. (2022). Utility of No-Charge Panel Genetic Testing for Inherited Retinal Diseases in a Real-World Clinical Setting. J. Vitreoretin. Dis..

[5] Taylor R.L., Parry N.R.A., Barton S.J., Campbell C., Delaney C.M., Ellingford J.M., Hall G., Hardcastle C., Morarji J., Nichol E.J. (2017). Panel-Based Clinical Genetic Testing in 85 Children with Inherited Retinal Disease. Ophthalmology.

[6] Li A.S., MacKay D., Chen H., Rajagopal R., Apte R.S. (2019). Challenges to Routine Genetic Testing for Inherited Retinal Dystrophies. Ophthalmology.

[7] Nykamp K., Anderson M., Powers M., Garcia J., Herrera B., Ho Y.-Y., Kobayashi Y., Patil N., Thusberg J., Westbrook M. (2017). Sherloc: A Comprehensive Refinement of the ACMG-AMP Variant Classification Criteria. Genet. Med..

[8] Blueprint Genetics Variant Classification—Genetic Variants. https://blueprintgenetics.com/variant-classification/.

[9] Invitae Invitae’s Method of Variant Classification. https://www.invitae.com/static/data/WhitePaper_Variant-Classification-Method.pdf.

[10] Klemm S. The Potential Benefits of Resolving Variants of Uncertain Significance in Genetic Testing. https://blog.invitae.com/the-potential-benefits-of-resolving-variants-of-uncertain-significance-in-genetic-testing-bec0bf94910.

[11] Kobayashi Y. How Genetics Experts Use Artificial Intelligence (AI) to Better Classify Gene Variants. https://blog.invitae.com/how-genetics-experts-use-artificial-intelligence-ai-to-better-classify-gene-variants-705ee18a0177.

[12] Blueprint Genetics VUS Classification Service. https://blueprintgenetics.com/vus-clarification/.

[13] Invitae Population Frequency Modeling. https://view.publitas.com/invitae/wp131_population-frequency-modeling_white-paper/page/1.

[14] Richards S., Aziz N., Bale S., Bick D., Das S., Gastier-Foster J., Grody W.W., Hegde M., Lyon E., Spector E. (2015). Standards and Guidelines for the Interpretation of Sequence Variants: A Joint Consensus Recommendation of the American College of Medical Genetics and Genomics and the Association for Molecular Pathology. Genet. Med..

[15] Pontikos N., Arno G., Jurkute N., Schiff E., Ba-Abbad R., Malka S., Gimenez A., Georgiou M., Wright G., Armengol M. (2020). Genetic Basis of Inherited Retinal Disease in a Molecularly Characterized Cohort of More Than 3000 Families from the United Kingdom. Ophthalmology.

[16] Echaniz-Laguna A., Nadjar Y., Béhin A., Biancalana V., Piraud M., Malfatti E., Laforêt P. (2019). Phosphoglycerate Kinase Deficiency: A Nationwide Multicenter Retrospective Study. J. Inherit. Metab. Dis..

[17] Cherry T.J., Yang M.G., Harmin D.A., Tao P., Timms A.E., Bauwens M., Allikmets R., Jones E.M., Chen R., De Baere E. (2020). Mapping the Cis-Regulatory Architecture of the Human Retina Reveals Noncoding Genetic Variation in Disease. Proc. Natl. Acad. Sci. USA.

[18] Mustafi D., Hisama F.M., Huey J., Chao J.R. (2022). The Current State of Genetic Testing Platforms for Inherited Retinal Diseases. Ophthalmol. Retina.

[19] Technology, Quality, & Scientific Contribution Faqs|for Providers|Invitae. https://www.invitae.com/us/provider-faqs/tech-and-quality.

[20] Panels. https://blueprintgenetics.com/tests/panels/.

[21] Huml A.M., Sullivan C., Figueroa M., Scott K., Sehgal A.R. (2020). Consistency of Direct to Consumer Genetic Testing Results Among Identical Twins. Am. J. Med..

