# Supplementary material for: Comparison of The Results of Sponsored Genetic Testing Panels for Inherited Retinal Diseases
Source: J Clin Med. 2024 May 26;13(11):3118. doi: 10.3390/jcm13113118 (PMC11172676; doi:10.3390/jcm13113118)
Supplement: Supplementary file 1 [file jcm-13-03118-s001.zip › jcm-3006811-supplementary.pdf]

Supplemental Table S1. List of Genes Tested by Both Invitae and Blueprint Genetics

ABCA4	*CA4*	*COL9A1*	*HK1*	*MKKS*	*PEX10*	*RCBTB1*	*TCTN1*	*VPS13B*
ABCC6	*CABP4*	*COL9A2*	*HMX1*	*MKS1*	*PEX11B*	*RD3*	*TCTN2*	*WDPCP*
ABHD12	*CACNA1F*	*COL9A3*	*IDH3A*	*MTTP*	*PEX12*	*RDH11*	*TCTN3*	*WDR19*
ACO2	*CACNA2D4*	*CRB1*	*IDH3B*	*MYO7A*	*PEX13*	*RDH12*	*TEAD1*	*WFS1*
ADAM9	*CAPN5*	*CRX*	*IFT140*	*NAGLU*	*PEX14*	*RDH5*	*TIMM8A*	*ZNF408*
ADAMTS18	*CC2D2A*	*CSPP1*	*IFT172*	*NDP*	*PEX16*	*REEP6*	*TIMP3*	*ZNF423*
ADGRV1	*CDH23*	*CTNNA1*	*IFT27*	*NEK2*	*PEX19*	*RGR*	*TMEM107*	*ZNF513*
ADIPOR1	*CDH3*	*CTSD*	*IFT81*	*NMNAT1*	*PEX2*	*RGS9*	*TMEM126A*	
AGBL5	*CDHR1*	*CWC27*	*IMPDH1*	*NPHP1*	*PEX26*	*RGS9BP*	*TMEM138*	
AHI1	*CEP164*	*CYP4V2*	*IMPG1*	*NPHP3*	*PEX3*	*RHO*	*TMEM216*	
AIPL1	*CEP19*	*DHDDS*	*IMPG2*	*NPHP4*	*PEX5*	*RIMS1*	*TMEM231*	
ALMS1	*CEP250*	*DHX38*	*INPP5E*	*NR2E3*	*PEX6*	*RLBP1*	*TMEM237*	
ARHGEF18	*CEP290*	*DRAM2*	*INVS*	*NR2F1*	*PEX7*	*ROM1*	*TMEM67*	
ARL13B	*CEP41*	*DTHD1*	*IQCB1*	*NRL*	*PHYH*	*RP1*	*TOPORS*	
ARL2BP	*CEP78*	*EFEMP1*	*JAG1*	*NYX*	*PLA2G5*	*RP1L1*	*TPP1*	
ARL3	*CEP83*	*ELOVL4*	*KCNJ13*	*OAT*	*PLK4*	*RP2*	*TRAF3IP1*	
ARL6	*CERKL*	*EMC1*	*KCNV2*	*OCA2*	*PNPLA6*	*RPE65*	*TREX1*	
ARMC9	*CHM*	*EXOSC2*	*KIAA0586*	*OFD1*	*POC1B*	*RPGR*	*TRIM32*	
ARSG	*CIB2*	*EYS*	*KIAA1549*	*OPA1*	*POMGNT1*	*RPGRIP1*	*TRPM1*	
ATF6	*CISD2*	*FAM161A*	*KIF11*	*OPA3*	*PPT1*	*RPGRIP1L*	*TSPAN12*	
ATOH7	*CLN3*	*FLVCR1*	*KIF7*	*OPN1SW*	*PRCD*	*RS1*	*TTC21B*	
B9D1	*CLN5*	*FRMD7*	*KIZ*	*OTX2*	*PRDM13*	*RTN4IP1*	*TTC8*	
BBIP1	*CLN6*	*FZD4*	*KLHL7*	*P3H2*	*PROM1*	*SAG*	*TTLL5*	
BBS1	*CLN8*	*GNAT1*	*LCA5*	*PAX2*	*PRPF3*	*SAMD11*	*TTPA*	
BBS10	*CLRN1*	*GNAT2*	*LRAT*	*PCDH15*	*PRPF31*	*SCLT1*	*TUB*	
BBS12	*CNGA1*	*GNB3*	*LRIT3*	*PCYT1A*	*PRPF4*	*SDCCAG8*	*TUBGCP4*	
BBS2	*CNGA3*	*GNPTG*	*LRP2*	*PDE6A*	*PRPF6*	*SEMA4A*	*TUBGCP6*	
BBS4	*CNGB1*	*GPR143*	*LRP5*	*PDE6B*	*PRPF8*	*SGSH*	*TULP1*	
BBS5	*CNGB3*	*GPR179*	*LZTFL1*	*PDE6C*	*PRPH2*	*SLC24A1*	*TYR*	
BBS7	*CNNM4*	*GRM6*	*MAK*	*PDE6D*	*PRPS1*	*SLC45A2*	*TYRP1*	
BBS9	*COL11A1*	*GUCA1A*	*MERTK*	*PDE6G*	*RAB28*	*SLC7A14*	*USH1C*	
BEST1	*COL11A2*	*GUCY2D*	*MFN2*	*PDE6H*	*RAX2*	*SNRNP200*	*USH1G*	
C1QTNF5	*COL18A1*	*HARS*	*MFRP*	*PDZD7*	*RBP3*	*SPATA7*	*USH2A*	
C8orf37	*COL2A1*	*HGSNAT*	*MFSD8*	*PEX1*	*RBP4*	*SPP2*	*VCAN*	

Supplemental Table S2. List of Genes Tested by Invitae Only and BG Only

**Invitae Only**	**BG Only**
ACBD5	*ABCD1*
ADAMTSL4	*AMACR*
ADGRA3	*ARR3*
AHR	*B9D2*
ASRGL1	*C21ORF2*
C10orf11	*C2ORF71*
C12orf65	*C5ORF42*
CCT2	*CEP104*
CFAP410	*CEP120*
CLCC1	*COQ2*
CLUAP1	*CPE*
CPLANE1	*CTC1*
DHX32	*CTNNB1*
DNAJC17	*DFNB31*
DSCAML1	*DNAJC5*
ERCC6	*DYNC2H1*
FBLN5	*ESPN*
FSCN2	*FDXR*
GDF6	*GRK1*
GNS	*ISPD*
GPR45	*KIAA0556*
GRN	*KIAA0753*
GUCA1B	*LAMA1*
HMCN1	*MMACHC*
IFT43	*MT-ATP6*
IFT74	*MT-ATP8*
IFT80	*MT-CO1*
IFT88	*MT-CO2*
ITM2B	*MT-CO3*
LYST	*MT-CYB*
MAPKAPK3	*MT-ND1*
MIR204	*MT-ND2*
MPDZ	*MT-ND3*
MTPAP	*MT-ND4*
NBAS	*MT-ND4L*
NEUROD1	*MT-ND5*

OR2W3	*MT-ND6*
PAX6	*MT-RNR1*
PCARE	*MT-RNR2*
PITP	*MT-TA*
POC5	*MT-TC*
RBP1	*MT-TD*
RP9	*MT-TE*
SIX6	*MT-TF*
SLC24A5	*MT-TG*
TMED7	*MT-TH*
TRNT1	*MT-TI*
UNC119	*MT-TK*
WDR34	*MT-TL1*
WHRN	*MT-TL2*
	MT-TM
	MT-TN
	MT-TP
	MT-TQ
	MT-TR
	MT-TS1
	MT-TS2
	MT- TT
	MT-TV
	MT-TW
	MT-TY
	MVK
	PANK2
	PDSS1
	PDSS2
	PISD
	PITPNM3
	SCAPER
	SLC25A46
	SRD5A3
	TUBB4B
	YME1L1

Supplemental Table S3. Frequency of Genes found to be pathogenic, likely pathogenic and VUS in Invitae Gene Panel

Invitae			
	Pathogenic	Likely Pathogenic	VUS
ABCA4	9	4	7
USH2A	8	1	13
EYS	4	0	10
CRB1	3	1	7
RP1	3	1	4
BBS7	3	1	3
ABCC6	3	0	1
CYP4V2	3	0	1
OCA2	3	0	1
MYO7A	2	0	12
VPS13B	2	0	11
ADGRV1	2	0	9
PROM1	2	1	5
CEP290	2	0	5
BEST1	2	0	3
ARSG	2	1	3
ROM1	2	1	7
CNGA3	2	0	1
FAM161A	2	0	1
TYR	2	0	1
LRP5	1	0	7
ALMS1	1	0	5
CNGB1	1	0	3
JAG1	1	0	3
PDE6B	1	0	3
ARMC9	1	0	2
CLN3	1	0	2
CSPP1	1	0	2
KIAA0586	1	0	2
NR2E3	1	1	2
RBP3	1	0	2
CNGB3	1	0	1
COL2A1	1	0	1
COL9A3	1	0	2

CWC27	1	0	2
ERCC6	1	0	1
IMPDH1	1	0	1
MFRP	1	0	1
MTTP	1	0	1
TMEM231	1	0	1
VCAN	0	0	8
CACNA2D4	0	0	8
RPGRIP1L	0	0	7
KIF7	0	0	6
PCDH15	0	0	5
CDH23	0	0	5
IFT140	0	0	5
ARHGEF18	0	0	4
ATF6	0	0	4
BBS9	0	0	4
CEP164	0	0	4
COL18A1	0	0	4
DHX38	0	0	4
FSCN2	0	0	4
GUCY2D	0	0	4
NPHP4	0	0	4
RIMS1	0	0	4
RP1L1	0	0	4
SAMD11	0	0	4
SDCCAG8	0	0	4
TUBGCP6	0	0	4
ADAM9	0	0	3
CDHR1	0	0	3
CEP250	0	0	3
CEP41	0	0	3
CNNM4	0	0	3
EMC1	0	0	3
HMCN1	0	0	3
IFT172	0	0	3
IFT81	0	0	3
INPP5E	0	0	3
MERTK	0	0	3
PEX5	0	0	3

PRPF3	0	0	4
RAX2	0	0	3
SEMA4A	0	0	3
SLC24A1	0	0	3
SLC7A14	0	0	3
TRPM1	0	0	3
TTC21B	0	0	3
TTLL5	0	0	3
TUB	0	0	3
TUBGCP4	0	0	3
UNC119	0	0	3
WDR19	0	0	3
ADGRA3	0	0	2
ARL13B	0	0	2
BBS1	0	0	2
BBS10	0	0	3
BBS12	0	0	2
CACNA1F	0	0	2
CLUAP1	0	0	2
COL9A1	0	0	2
CPLANE1	0	0	2
DRAM2	0	0	2
DTHD1	0	0	2
GNAT1	0	0	2
GNAT2	0	0	2
GPR179	0	0	2
GUCA1B	0	0	2
KCNJ13	0	0	2
KCNV2	0	0	2
KIAA1549	0	0	2
KIF11	0	0	2
LRIT3	0	0	2
MKKS	0	0	2
NPHP1	0	0	3
NPHP3	0	0	2
P3H2	0	0	2
PDZD7	0	0	2
PITPNM3	0	0	2
PLK4	0	0	2

RBP4	0	0	2
RDH11	0	0	2
SAG	0	1	2
USH1G	0	0	2
WFS1	0	0	3
WHRN	0	0	2
ZNF513	0	0	2
ACBD5	0	0	1
ADAMTS18	0	0	1
ADIPOR1	0	0	1
AGBL5	0	0	1
AHR	0	0	1
AIPL1	0	0	1
ASRGL1	0	0	1
BBS2	0	0	1
CA4	0	0	2
CAPN5	0	0	1
CEP83	0	0	1
CERKL	0	0	1
CFAP410	0	0	1
CIB2	0	0	1
CLN5	0	0	1
CNGA1	0	0	1
COL11A1	0	0	1
COL11A2	0	0	1
CRX	0	0	1
CTNNA1	0	0	1
DNAJC17	0	0	1
EFEMP1	0	0	1
ELOVL4	0	0	1
FBLN5	0	0	1
FLVCR1	0	0	1
FRMD7	0	0	1
FZD4	0	0	1
GDF6	0	0	1
GNPTG	0	0	1
GPR143	0	0	1
GPR45	0	0	1
GRM6	0	0	1

GUCA1A	0	0	1
HGSNAT	0	0	1
HMX1	0	0	1
IDH3B	0	0	1
IMPG1	0	0	1
IQCB1	0	0	1
LRP2	0	0	1
LYST	0	0	1
MAPKAPK3	0	0	1
MFN2	0	0	1
MFSD8	0	0	1
MKS1	0	0	1
MPDZ	0	0	1
MTPAP	0	0	1
NAGLU	0	0	1
NBAS	0	0	1
NMNAT1	0	0	1
PDE6A	0	0	1
PEX10	0	0	1
PEX12	0	0	1
PEX19	0	0	1
PEX3	0	0	1
PHYH	0	0	1
POC1B	0	0	1
PRCD	0	0	1
PRPF31	0	0	1
PRPF6	0	0	1
PRPF8	0	0	1
REEP6	0	0	1
RGR	0	0	1
RGS9	0	0	1
RPE65	0	0	1
RPGR	0	0	1
RS1	0	0	1
SPATA7	0	0	1
TCTN2	0	0	1
TCTN3	0	0	1
TIMP3	0	0	1
TMEM216	0	0	1

TMEM237	0	0	1
TMEM67	0	0	1
TPP1	0	0	1
TRAF3IP1	0	0	1
TRIM32	0	0	1
TRNT1	0	0	1
TTPA	0	0	1
WDPCP	0	0	1
WFS	0	0	3
ZNF408	0	0	1

Supplemental Table S4. Frequency of Genes found to be pathogenic, likely pathogenic and VUS in Blueprint Genetics Panel

	Pathogenic	Likely Pathogenic	VUS
ABCA4	10	4	10
RHO	6	0	1
EYS	4	2	9
USH2A	4	0	10
CLN3	3	0	1
RPGR	3	1	3
CERKL	2	1	1
RLBP1	2	1	0
ABCC6	1	0	1
AIPL1	1	0	0
ALMS1	1	0	0
BBS12	1	0	1
BBS2	1	0	1
BBS7	1	0	1
BEST1	1	0	0
CDHR1	1	0	1
CNNM4	1	0	0
CRX	1	0	0
CYP4V2	1	0	1
FLVCR1	1	0	1
HGSNAT	1	1	0
HK1	1	0	1
MFRP	1	0	0
MMACHC	1	0	0
MT-ATP6	1	0	0
MT-ND1	1	0	0
MYO7A	1	2	0
NR2E3	1	1	2
OCA2	1	0	0
PCDH15	1	0	0
PEX11B	1	0	0
PEX12	1	0	0

RP1	1	0	1
RP1L1	1	0	4
RP2	1	0	0
TMEM231	1	0	2
TREX1	1	0	0
TTC21B	1	0	0
RCBTB1	1	1	1
KIAA1549	0	0	7
AGBL5	0	0	5
CNGB1	0	0	4
IFT140	0	0	4
PDE6A	0	1	4
CACNA1F	0	0	3
GUCY2D	0	0	3
IFT172	0	0	4
PCARE	0	0	3
PRPH2	0	0	3
RBP3	0	0	3
RIMS1	0	0	3
SAG	0	2	3
ARHGEF18	0	0	2
CAPN5	0	0	2
CNGA1	0	1	2
CRB1	0	0	2
DHX38	0	0	2
GUCA1A	0	0	2
MFSD8	0	0	2
PDE6B	0	3	3
RPGRIP1	0	1	2
SEMA4A	0	0	2
SLC7A14	0	0	2
TULP1	0	0	2
ADAM9	0	0	1
ADGRV1	0	2	2
ARSG	0	0	1
ATF6	0	0	1

BBS10	0	0	1
C2ORF71	0	0	1
CA4	0	0	1
CABP4	0	0	1
CACNA2D4	0	0	1
CC2D2A	0	0	1
CEP250	0	0	1
CFAP418	0	0	1
CLRN1	0	0	1
COL9A1	0	0	1
COL9A3	0	1	1
CPLANE1	0	0	1
CWC27	0	0	1
DFNB31	0	0	1
DYNC2H1	0	0	1
FAM161A	0	0	1
GRK1	0	0	1
IDH3B	0	0	1
IFT17	0	0	4
IMPG1	0	0	1
JAG1	0	0	1
KCNV2	0	0	1
KIZ	0	0	1
LAMA1	0	0	1
LCA5	0	0	1
MAK	0	0	1
MERTK	0	0	1
MFN2	0	0	1
MTTP	0	0	1
NEK2	0	0	1
OPN1SW	0	0	1
OTX2	0	0	1
P3H2	0	0	1
PANK2	0	0	1
PNPLA6	0	0	1
POC1B	0	0	1

POMGNT1	0	0	1
PROM1	0	0	1
RAX2	0	0	1
RGR	0	0	1
TUB	0	2	1
USH1C	0	0	1
VCAN	0	0	1
VPS13B	0	1	1
WDR19	0	0	1
